# MRI Findings of Ovarian Carcinosarcoma: A Retrospective Comparison With Endometrioid Carcinoma

**DOI:** 10.7759/cureus.104738

**Published:** 2026-03-05

**Authors:** Akane Tashiro, Tomohisa Okuma, Taiki Yamaguchi, Takashi Honjo, Nahoko Inoue, Suzuka Okajima, Makoto Murakami, Tomoyuki Ichimura

**Affiliations:** 1 Radiodiagnosis, Osaka City General Hospital, Osaka, JPN; 2 Obstetrics and Gynecology, Osaka City General Hospital, Osaka, JPN; 3 Gynecology, Osaka City General Hospital, Osaka, JPN

**Keywords:** cancer, magnetic resonance imaging, malignant mixed epithelial and mesenchymal tumor, ovarian carcinosarcoma, ovarian endometrioid carcinoma

## Abstract

Purpose

Ovarian carcinosarcoma is a rare and highly aggressive ovarian malignancy composed of epithelial and mesenchymal components, and its preoperative diagnosis remains challenging because of nonspecific imaging features. This study aimed to identify MRI findings useful for differentiating ovarian carcinosarcoma from ovarian endometrioid carcinoma by comparing their imaging characteristics.

Materials and methods

We retrospectively reviewed patients who underwent pelvic MRI between January 2002 and December 2025 for suspected malignant ovarian tumors and were subsequently diagnosed with ovarian carcinosarcoma or ovarian endometrioid carcinoma on postoperative histopathology. Patient age and MRI findings were analyzed. Evaluated MRI features included tumor size, laterality, morphology, margin characteristics, tumor architecture, signal heterogeneity on T2-weighted images, morphology of solid components, presence of a stained-glass appearance, mille-feuille-like layered pattern, hemorrhage, enhancement characteristics, necrosis, lymphadenopathy, ascites, and peritoneal dissemination. Apparent diffusion coefficient (ADC) values of solid tumor components were measured. Imaging findings were compared between groups using Fisher’s exact test and the Mann-Whitney U test.

Results

Among 12 cases of ovarian carcinosarcoma, the maximum tumor diameter ranged from 5.7 to 21.5 cm (mean, 11.6 ± 4.8 cm). Tumors were unilateral in 11 cases. An irregular tumor margin was observed in 6 cases, solid-dominant components in 7 cases, nodular solid components in 12 cases, the presence of a mille-feuille sign in 2 cases, lymphadenopathy in 6 cases, peritoneal dissemination in 8 cases, and ascites in 9 cases. The mean ADC value of the solid component in ovarian carcinosarcoma was 0.952 ± 0.186 × 10⁻³ mm²/s. Significant differences between ovarian carcinosarcoma and ovarian endometrioid carcinoma were observed for patient age (67.6 years vs. 56.9 years, p=0.003), margin irregularity (6/12:50% vs. 3/45:6.7%, p=0.002), predominance of solid components (7/12:58.3% vs. 11/45:24.4%, p=0.037), nodular morphology of solid portions (12/12:100% vs. 25/45:55.6%, p=0.005), presence of the mille-feuille sign (2/12:16.7% vs. 0/45:0%, p=0.041), and peritoneal dissemination (8/12:66.7% vs. 9/45:20%, p=0.004). Laterality, overall tumor shape, the presence of hemorrhagic components, and mean ADC value did not differ significantly between the two groups.

Conclusion

On MRI, ovarian carcinosarcoma tends to present as a mass with irregular margins, predominantly nodular solid components with partial cystic components, and frequent peritoneal dissemination. Recognition of these characteristic findings may help differentiate ovarian carcinosarcoma from ovarian endometrioid carcinoma and improve preoperative diagnostic accuracy in patients with malignant ovarian tumors.

## Introduction

Ovarian carcinosarcoma, also referred to as ovarian malignant mixed Müllerian tumor, is a rare and highly malignant tumor composed of both epithelial carcinoma and sarcomatous mesenchymal components. It predominantly affects postmenopausal women and typically presents with nonspecific clinical symptoms, including abdominal distension, lower abdominal pain, and genital bleeding. As a result, the disease is often diagnosed at an advanced stage. The standard treatment is cytoreductive surgery followed by chemotherapy, which has been reported to improve survival; however, ovarian carcinosarcoma is generally considered more resistant to chemotherapy than other types of ovarian malignancies and is associated with an overall poor prognosis [1-5].

Accurate preoperative identification of ovarian carcinosarcoma can guide surgical planning and prognosis counseling. Ovarian carcinosarcoma has been limited to case reports or small case series [6,7]. However, these findings are nonspecific and may overlap substantially with those of other epithelial ovarian malignancies.

Both ovarian carcinosarcoma and ovarian endometrioid carcinoma are known to present as masses containing both solid and cystic components, often including areas of high signal intensity on T1-weighted images reflecting hemorrhage, and their imaging findings may partially overlap. Previous studies have already compared the imaging findings of ovarian carcinosarcoma with those of metastatic tumors and high-grade serous carcinoma [8,9]. However, no previous studies have compared the imaging findings of ovarian carcinosarcoma with those of ovarian endometrioid carcinoma. Therefore, the purpose of this study was to determine MRI characteristics that reliably distinguish ovarian carcinosarcoma from ovarian endometrioid carcinoma preoperatively.

## Materials and methods

The study protocol was approved by the Institutional Review Board of Osaka City General Hospital (Approval No. 2511117). Informed consent was obtained in the form of opt-out by displaying a notice about the study on the hospital website, providing patients with the opportunity to decline participation. We retrospectively identified patients who underwent pelvic MRI examinations for the purpose of lesion characterization and staging between January 2002 and December 2025, where a malignant ovarian tumor was clinically suspected. Using the electronic medical record system, patients with a definitive postoperative histopathologic diagnosis of ovarian carcinosarcoma or ovarian endometrioid carcinoma were identified. Pathological diagnoses were based on pathology reports, and the pathologists were blinded to the imaging findings. We retrospectively analyzed the age of the patient and MRI findings. Participants who withdrew consent were excluded. In addition, cases not diagnosed as ovarian carcinosarcoma or ovarian endometrioid carcinoma and those without available imaging data were also excluded.

MRI protocol

MRI examinations were performed using 1.5-T or 3.0-T scanners (Achieva, Achieva dStream; Philips Medical Systems; Magnetom Harmony, Skyra, Vida fit; Siemens Healthineers). The evaluated imaging sequences included spin-echo T1-weighted imaging, fast spin-echo T2-weighted imaging, single-shot fast spin-echo T2-weighted imaging, fat-suppressed T1-weighted imaging, diffusion-weighted imaging (DWI), and fat-suppressed spin-echo contrast-enhanced T1-weighted imaging. Imaging parameters were as follows: slice thickness, 4-6 mm; interslice gap, 0.4-0.6 mm; matrix size, 320 × 320 to 512 × 512; field of view, 25-40 cm. The repetition time/echo time was 435-573/9-13 msec for T1-weighted imaging, 3500-8060/91-109 msec for T2-weighted imaging, 1200-300/75-110 msec for single-shot fast spin-echo T2-weighted imaging, 524-753/8-15 msec for fat-suppressed T1-weighted imaging, and 2700-3055/70-83 msec for DWI. DWI was performed using a b-value of 1000 s/mm². Contrast-enhanced MRI was performed using gadoterate meglumine (GE HealthCare Pharma Japan) at a dose of 0.2 mL/kg or gadobutrol (Gadovist, Bayer Pharma Japan) at a dose of 0.1 mL/kg.

Image analysis

The following imaging findings were evaluated: maximum tumor diameter; laterality (unilateral or bilateral); tumor shape (lobulated or oval shape, presence of irregular margins); tumor structure (solid-dominant or cystic-dominant); internal signal characteristics (signal homogeneity on T2-weighted images and the presence of a “stained-glass” appearance defined as mixed areas of high and low signal intensity within cystic components on T2-weighted images); morphology of solid components (nodular or papillary); presence of a “mille-feuille-like” layered appearance, characterized by alternating thin layers of differing signal intensities on T2-weighted images; presence of intratumoral hemorrhage showing high signal intensity on T1-weighted images and low signal intensity on T2-weighted images with fluid-fluid levels; presence and homogeneity of enhancement of the solid components; presence of internal necrosis; and presence of lymphadenopathy, ascites, or peritoneal dissemination [8,9]. For quantitative analysis, circular regions of interest were placed on the solid tumor areas showing the lowest apparent diffusion coefficient (ADC) values while avoiding obvious necrosis or hemorrhage, and the mean ADC value was measured. The region of interest (ROI) was placed on the solid component by referencing the T2-weighted images side-by-side, avoiding areas of necrosis or hemorrhage, and selecting the region that showed the highest signal on DWI and the lowest signal on the ADC map, using a circular ROI of at least 50 mm². Imaging assessment was independently performed by two radiologists. In cases of disagreement, a final consensus was reached through discussion.

Statistical analysis

Patient age and imaging findings were compared between ovarian carcinosarcoma and ovarian endometrioid carcinoma. Imaging findings were analyzed using Fisher’s exact test. Continuous variables (age, tumor size, and mean ADC values) were analyzed using the Mann-Whitney U test. A p-value of less than 0.05 was considered statistically significant. We calculated the inter-reader concordance rate and the kappa value for morphology and imaging findings. Statistical analyses were performed using GraphPad Prism 8 for macOS (version 8.4.3, Dotmatics, Boston, Massachusetts).

## Results

**Figure 1:**
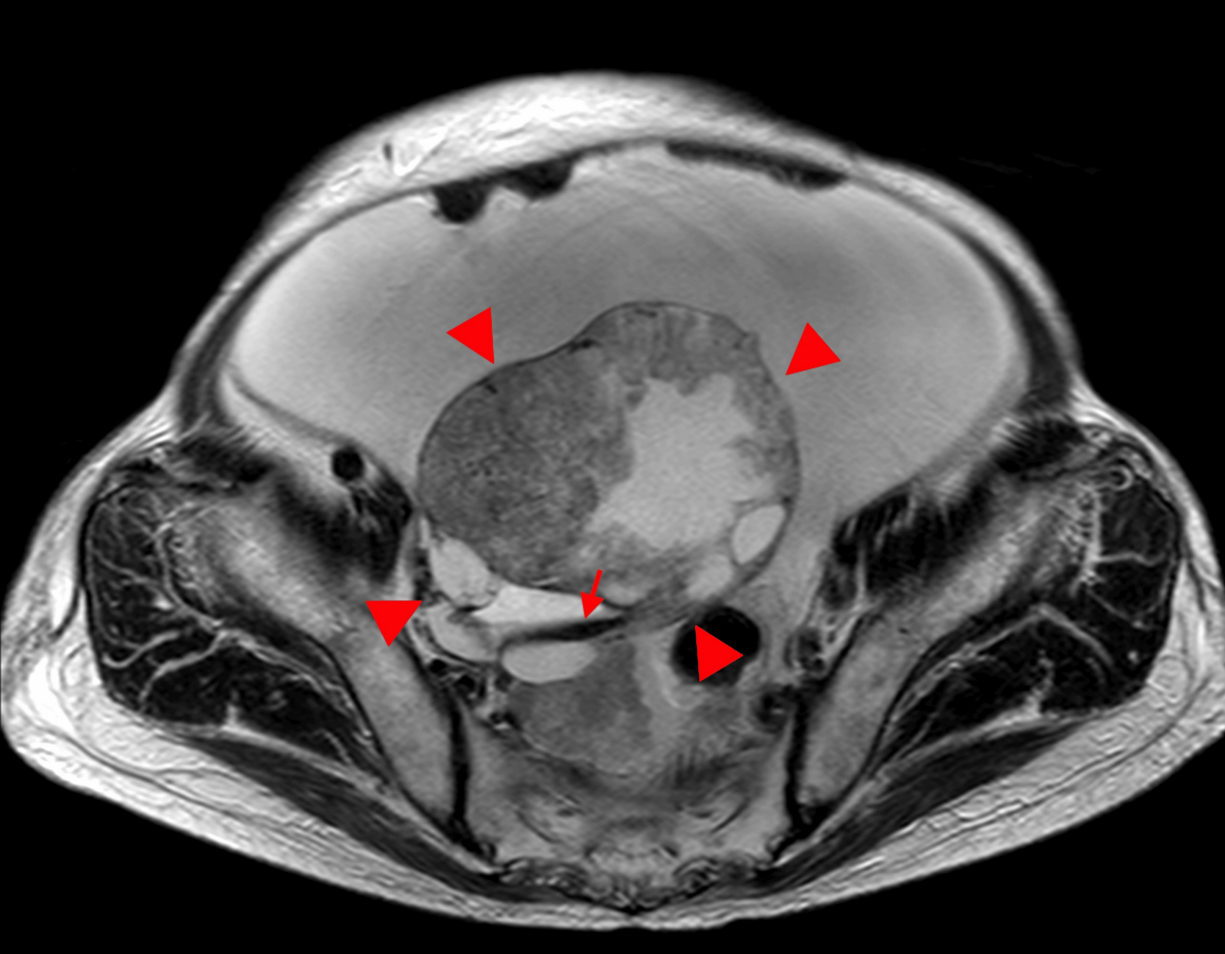
MRI in a 77-year-old woman with ovarian carcinosarcoma of the right ovary A large tumor measuring up to 12 cm in diameter is observed, consisting of broad-based, concentric solid components with heterogeneous signal intensity on the axial T2-weighted image and associated cystic components (arrowhead). The posterior margin is irregular and contains a fluid-fluid level suggestive of hemorrhage (arrow).

**Figure 2:**
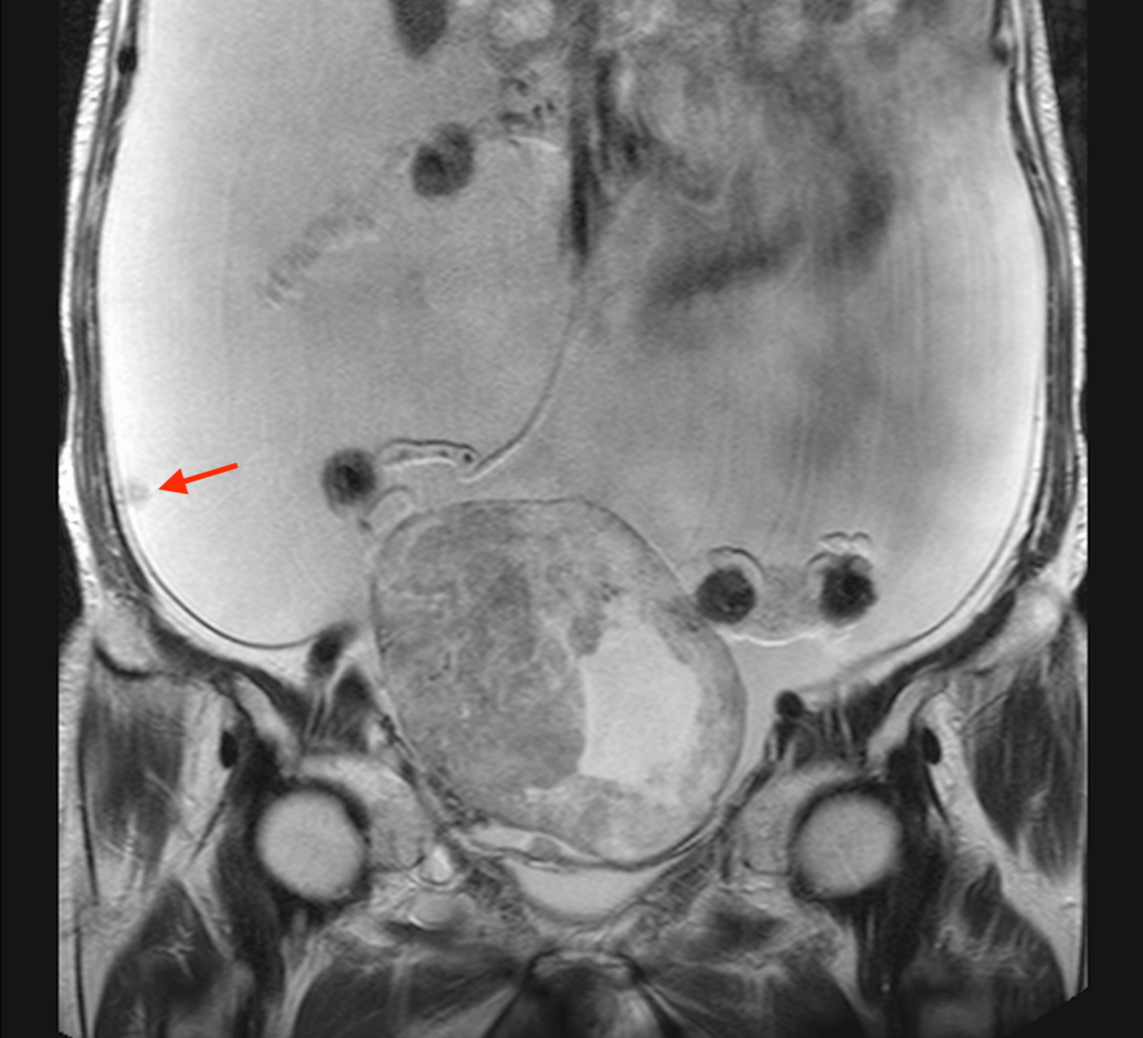
This is the same patient as shown in Figure 1. The coronal T2-weighted image reveals massive ascites and peritoneal dissemination (arrow).

**Figure 3:**
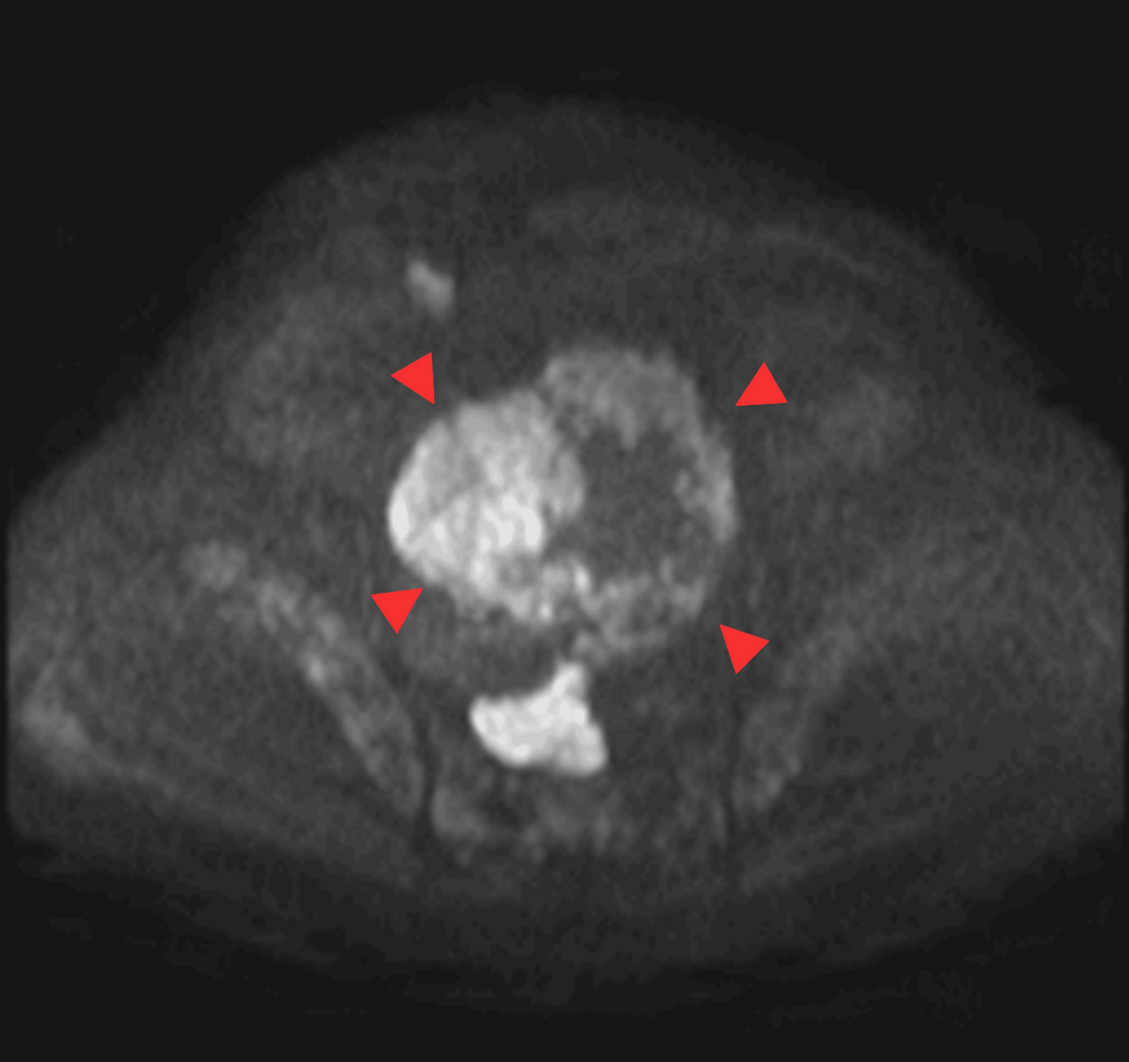
This is the same patient as shown in Figure 1. On the diffusion-weighted imaging, the solid component shows high signal intensity (arrowhead).

**Figure 4:**
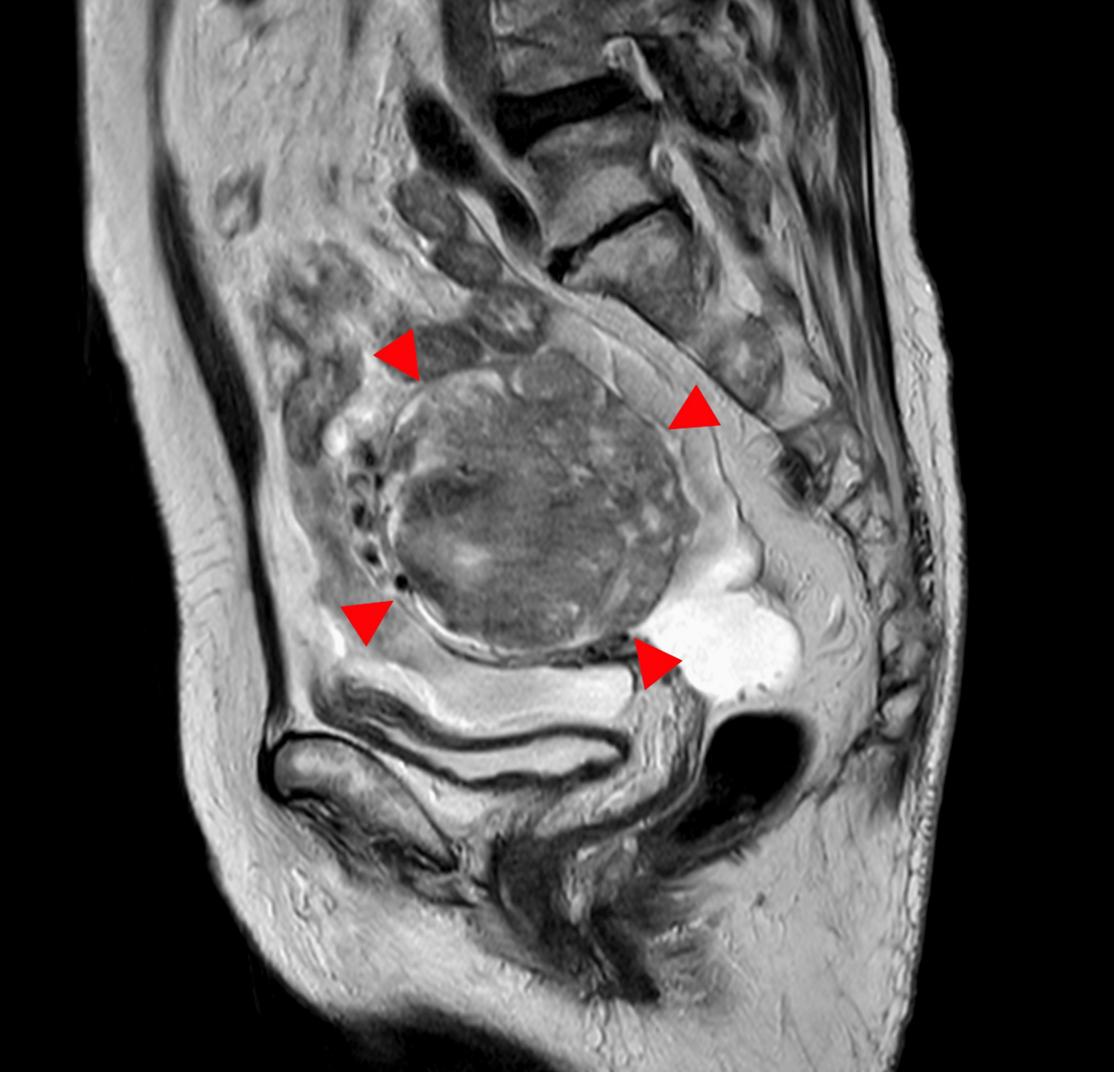
MRI in an 80-year-old woman with ovarian carcinosarcoma of the right ovary The sagittal T2-weighted image demonstrates a well-defined solid mass with heterogeneous signal intensity and partial cystic components (arrowhead). The tumor measures 7.2 cm in diameter.

**Figure 5:**
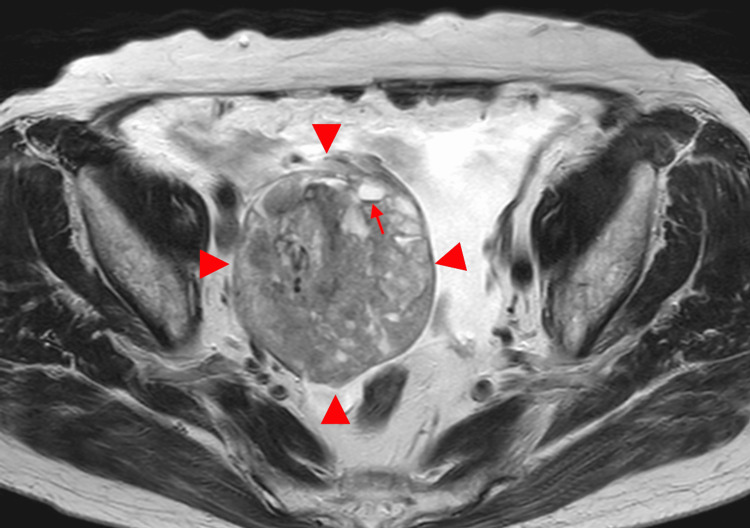
This is the same patient as shown in Figure 4. The axial T2-weighted image demonstrates a well-defined solid mass with heterogeneous signal intensity and partial cystic components (arrowhead). Areas of low signal intensity and fluid levels are noted within the mass (arrow).

**Figure 6:**
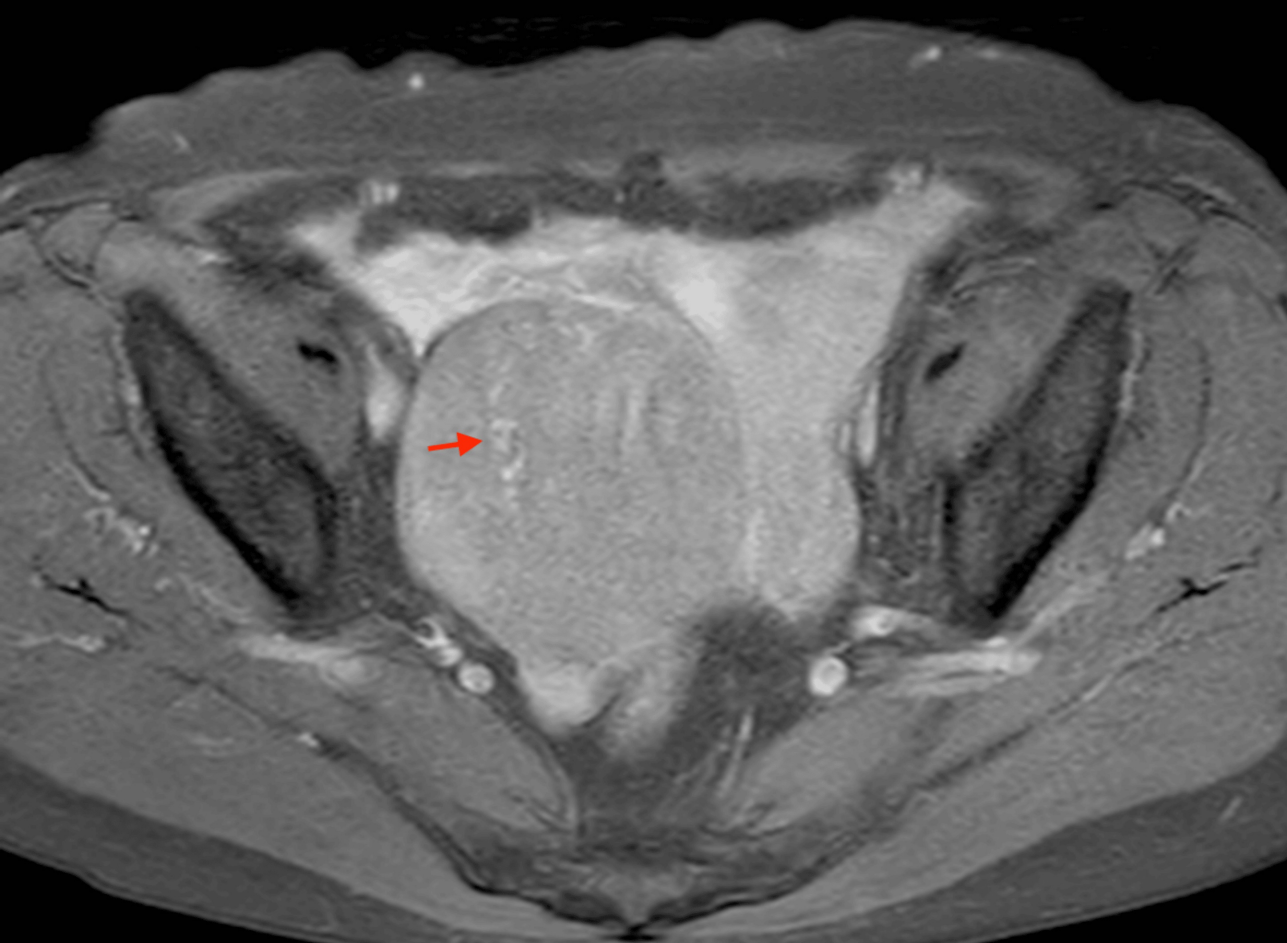
This is the same patient as shown in Figure 4. The axial fat-suppressed T1-weighted image shows high signal intensity, suggestive of hemorrhage (arrow).

**Figure 7:**
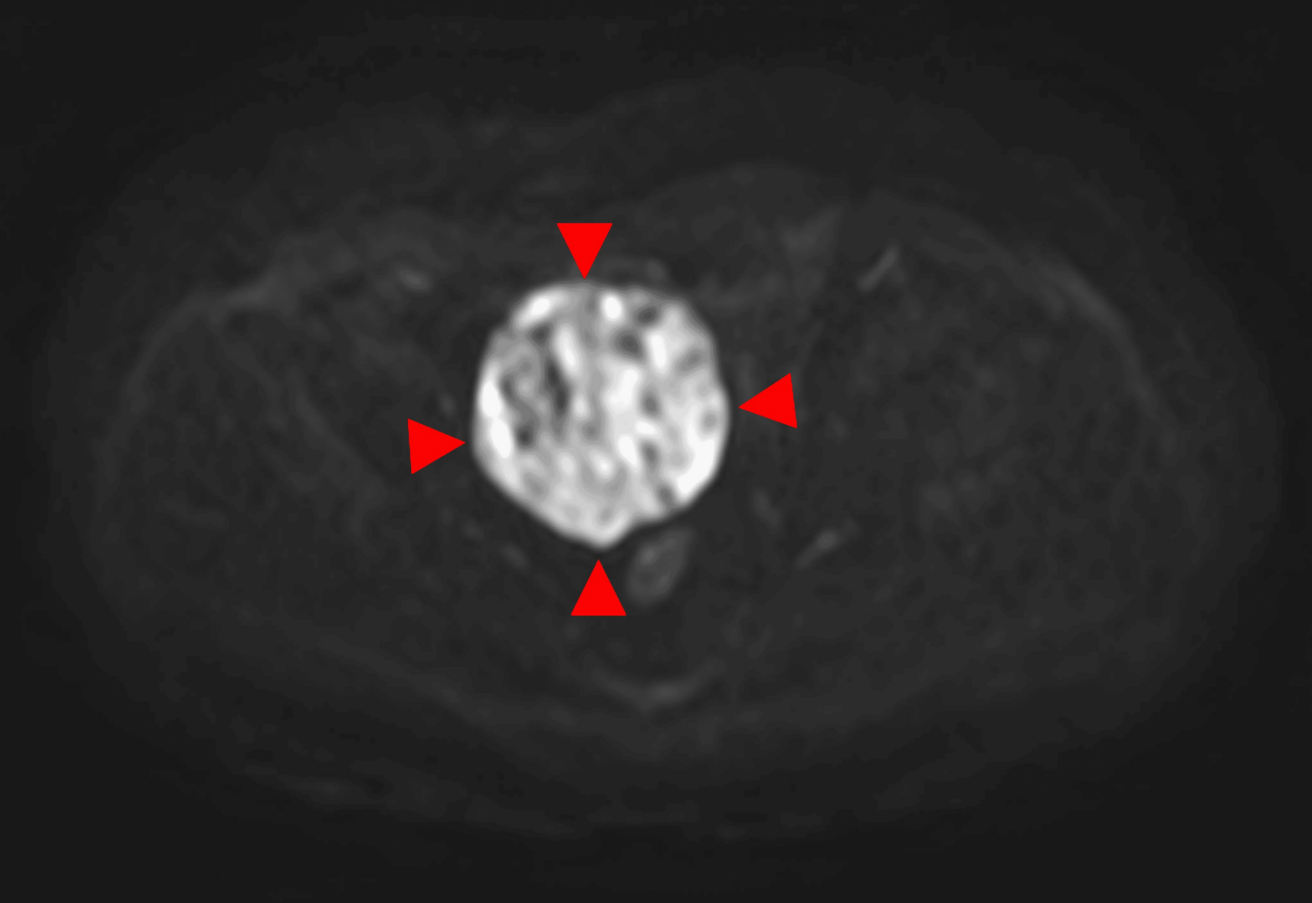
This is the same patient as shown in Figure 4. The axial diffusion-weighted imaging demonstrates high signal intensity in the solid component (arrowhead).

**Figure 8:**
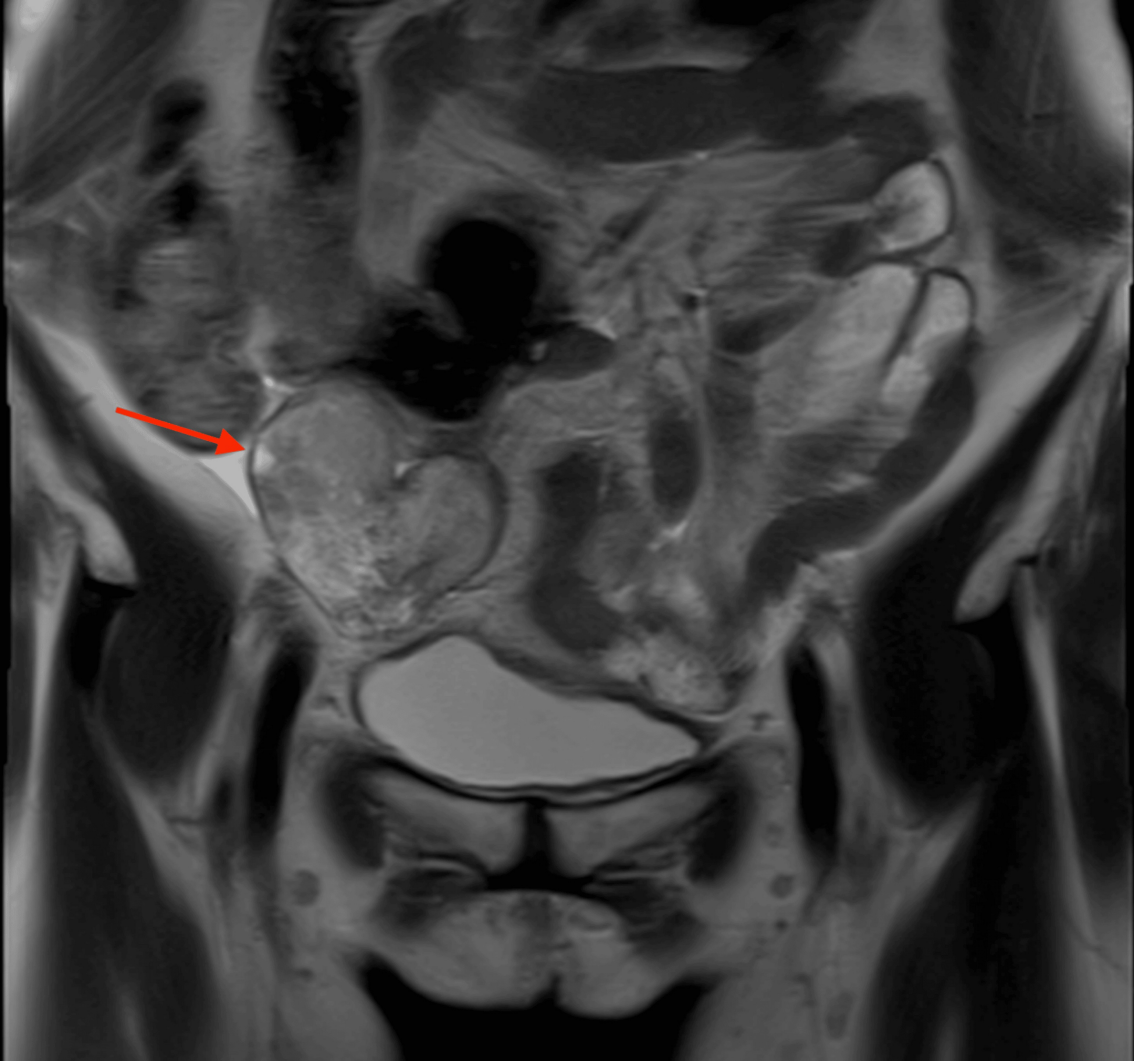
MRI in a 62-year-old woman with ovarian carcinosarcoma of the right ovary A heart-shaped tumor with well-defined margins and predominantly solid components is observed on the coronal T2-weighted image (arrow). The tumor measures 5.7 cm in diameter.

**Figure 9:**
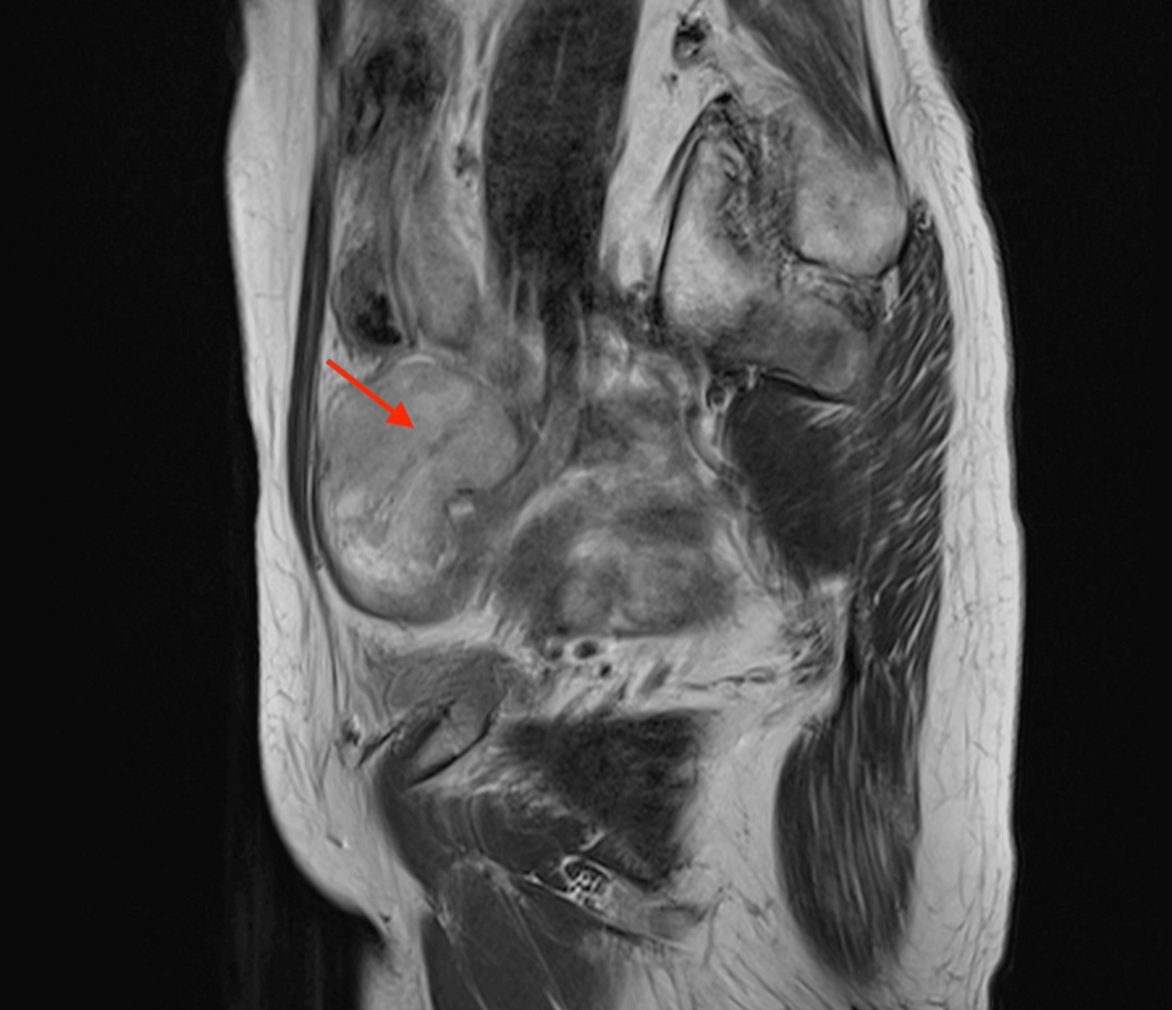
This is the same patient as shown in Figure 8. On the sagittal T2-weighted image, the lesion demonstrates heterogeneous signal intensity with alternating high- and low-signal layers, consistent with the mille-feuille sign (arrow).

**Figure 10:**
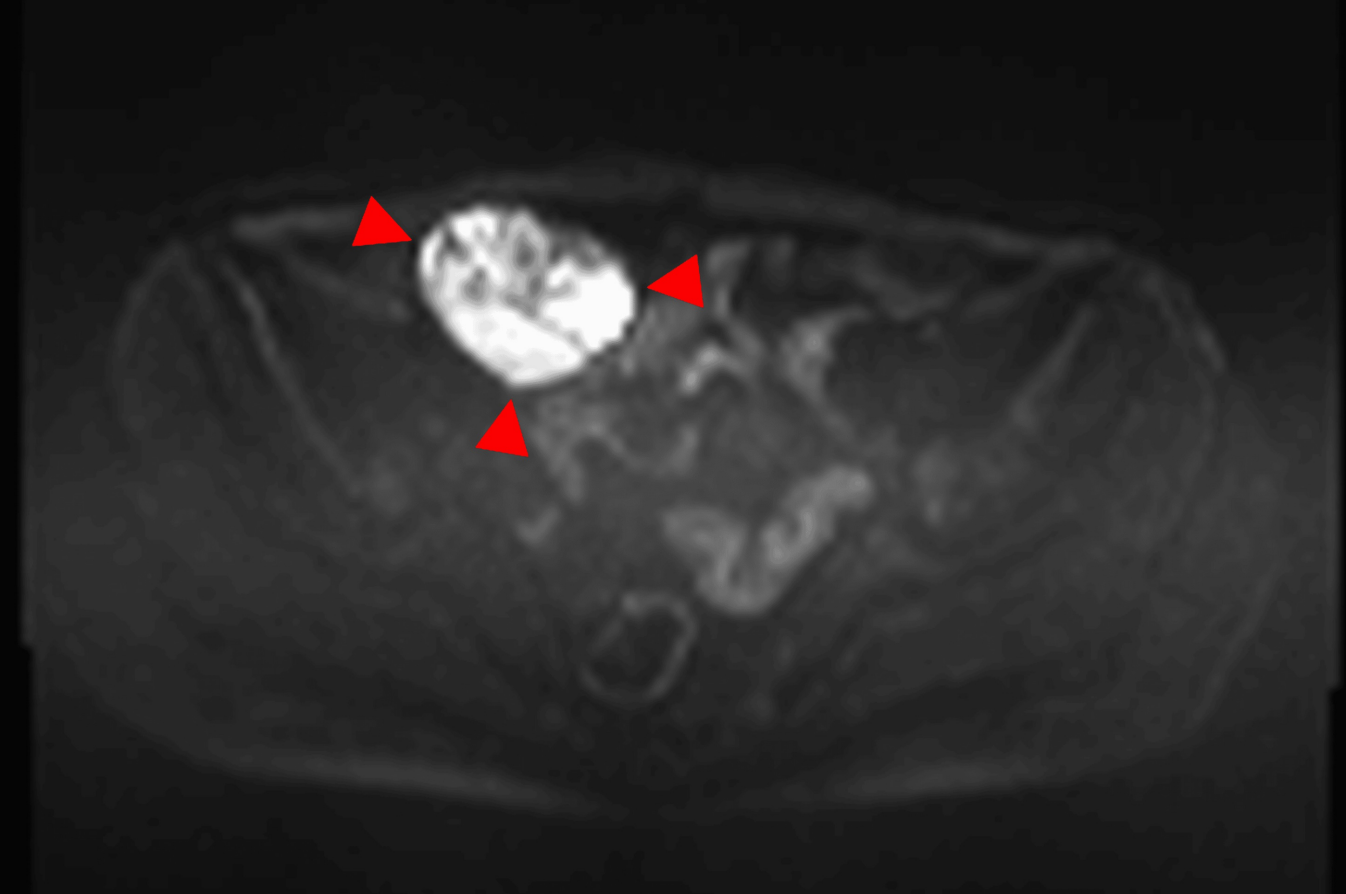
This is the same patient as shown in Figure 8. The diffusion-weighted imaging shows high signal intensity (arrowhead).

**Figure 11:**
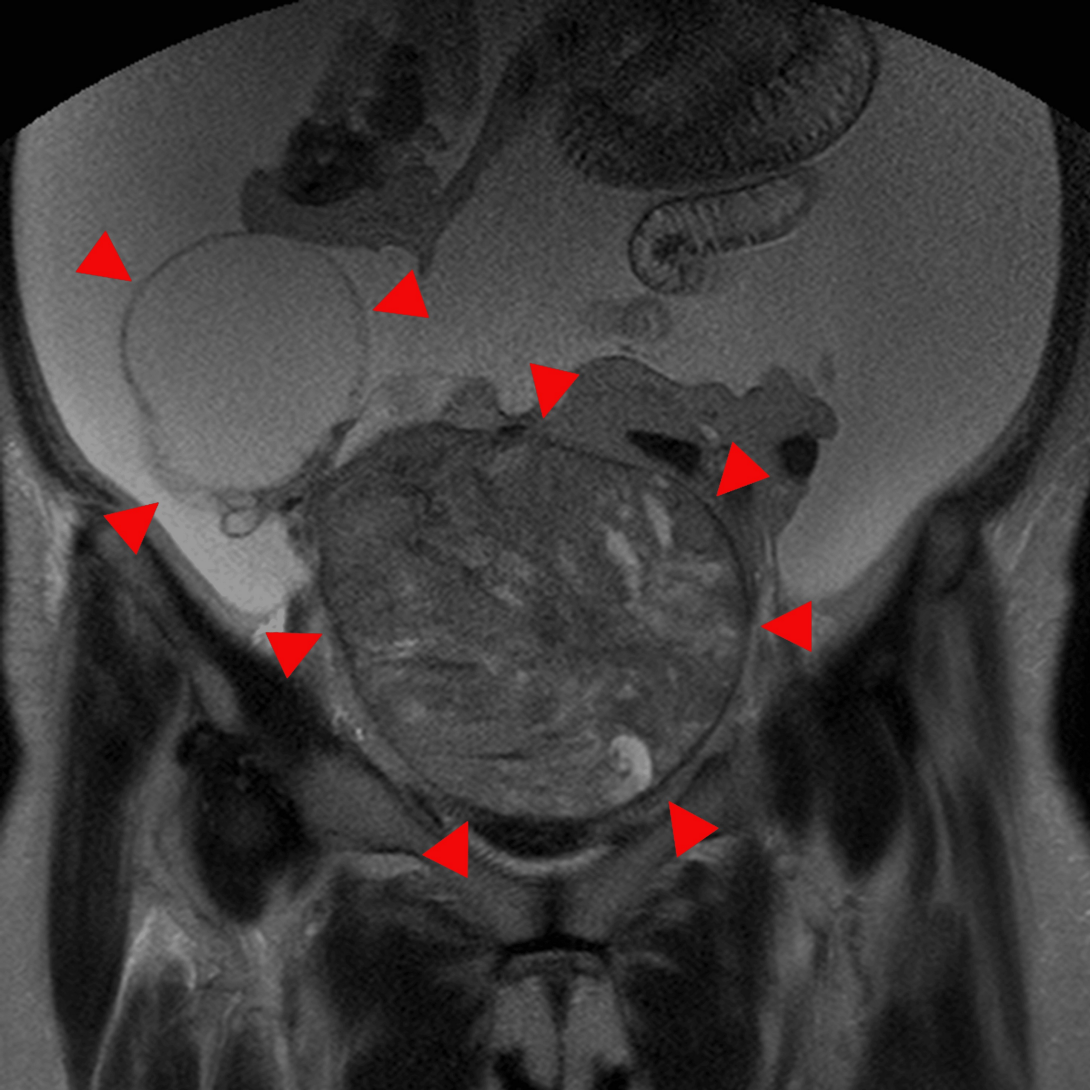
MRI in a 66-year-old woman with ovarian carcinosarcoma of the right ovary The coronal T2-weighted image shows a mass containing both solid and cystic components, accompanied by ascites (arrowhead). The tumor measures 21.5 cm in diameter.

**Figure 12:**
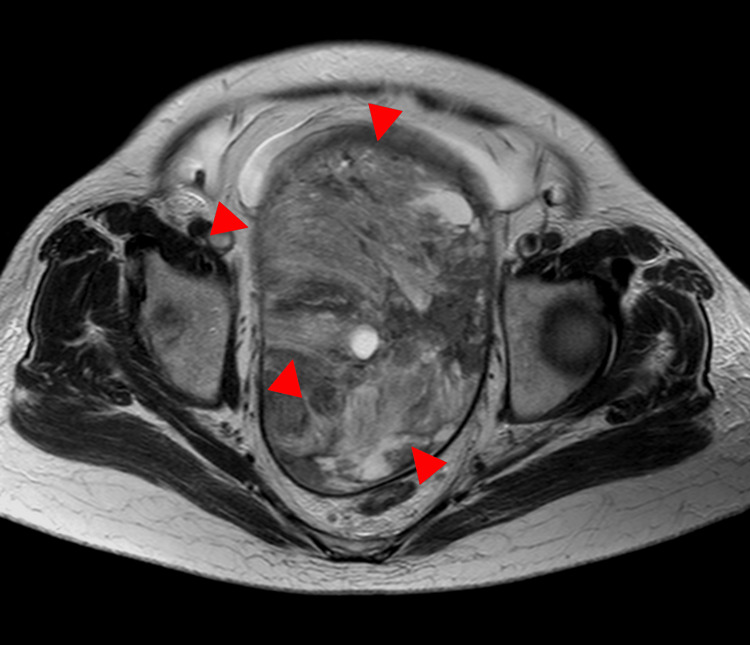
This is the same patient as shown in Figure 11. On the axial T2-weighted image, the solid component demonstrates heterogeneous signal intensity with a layered appearance, consistent with the mille-feuille sign (arrowhead).

**Figure 13:**
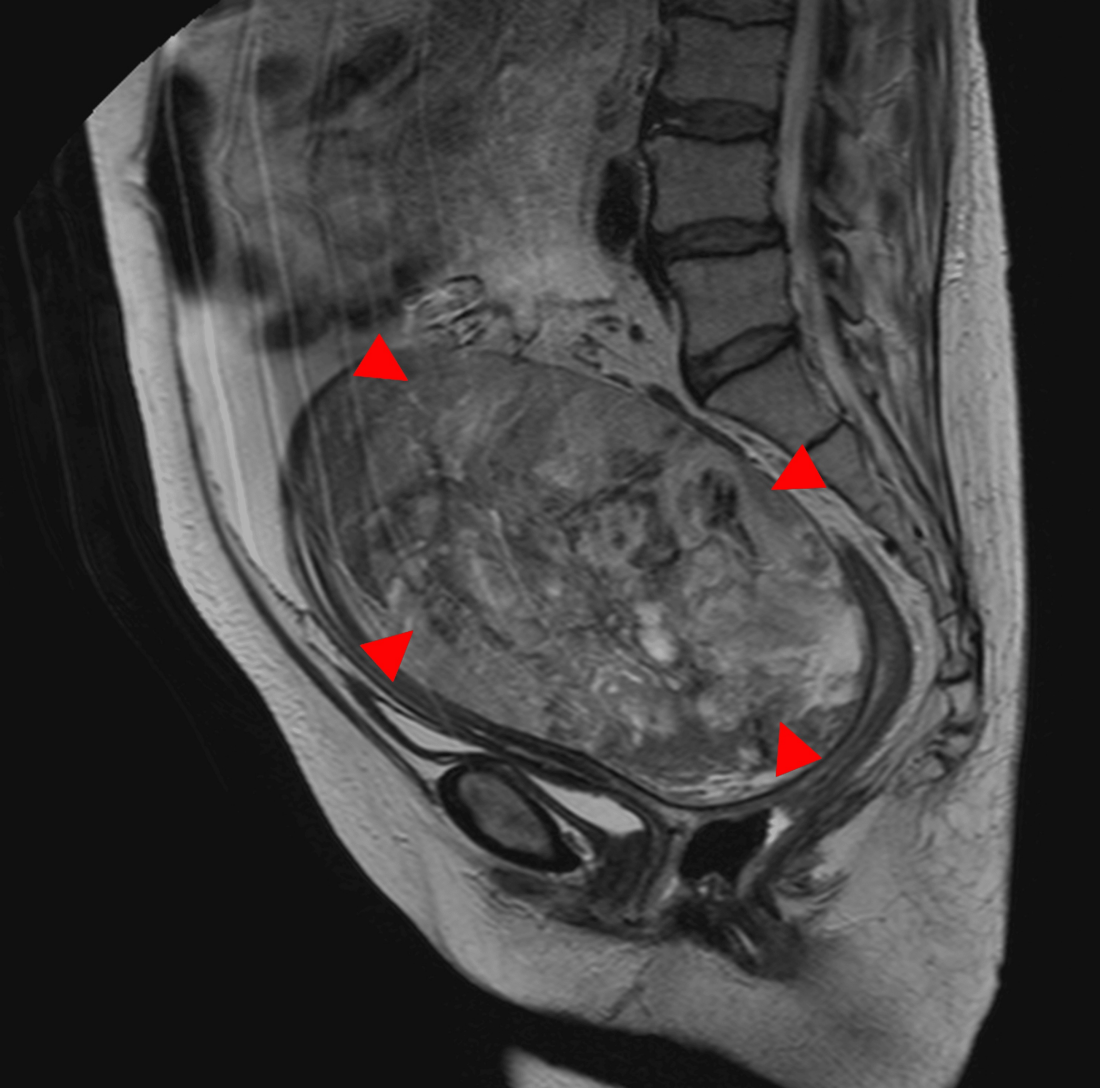
This is the same patient as shown in Figure 11. On the sagittal T2-weighted image, the solid component demonstrates heterogeneous signal intensity with a layered appearance, consistent with the mille-feuille sign (arrowhead).

**Figure 14:**
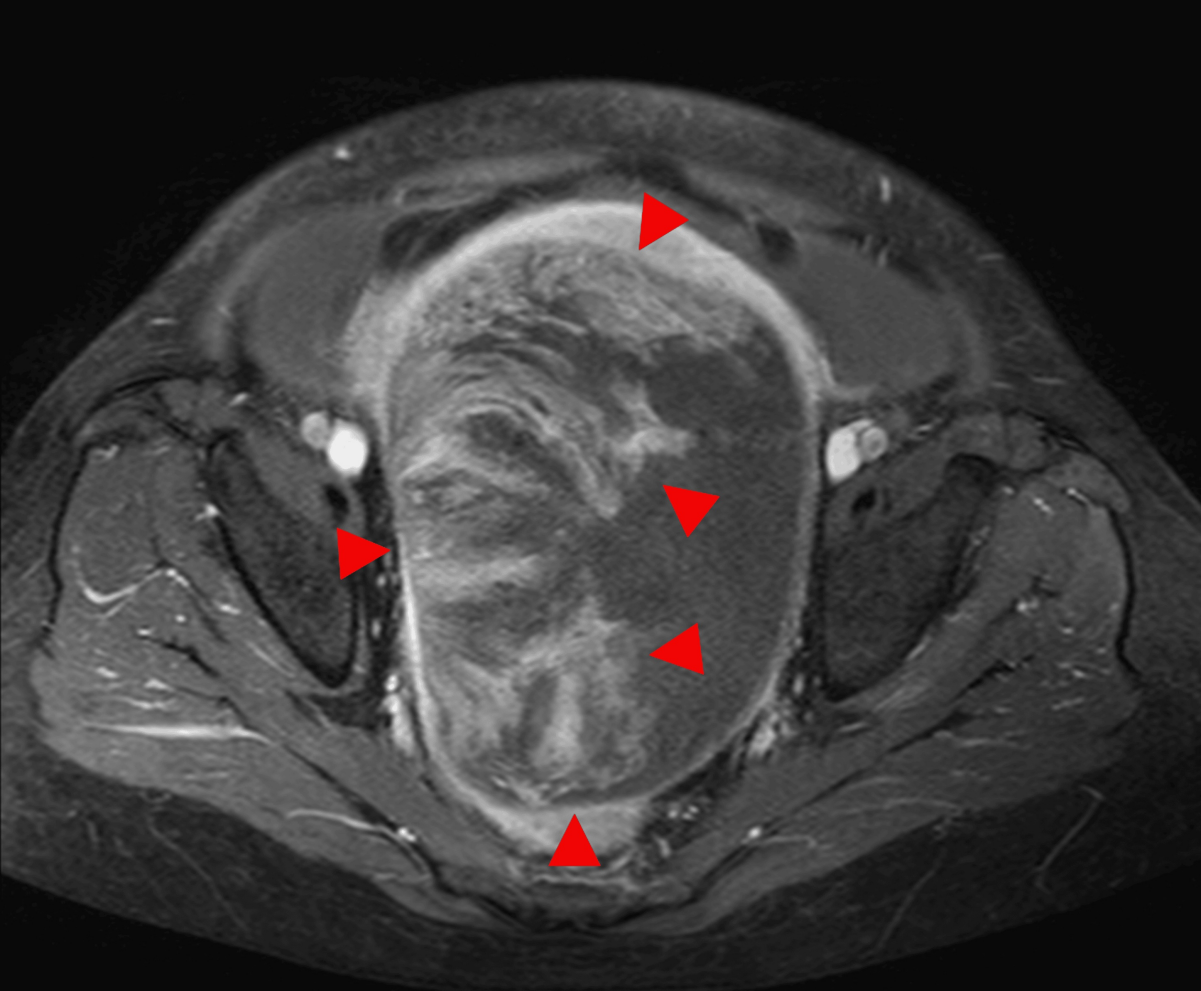
This is the same patient as shown in Figure 11. The contrast-enhanced image shows a corresponding layered enhancement pattern (arrowhead).

**Figure 15:**
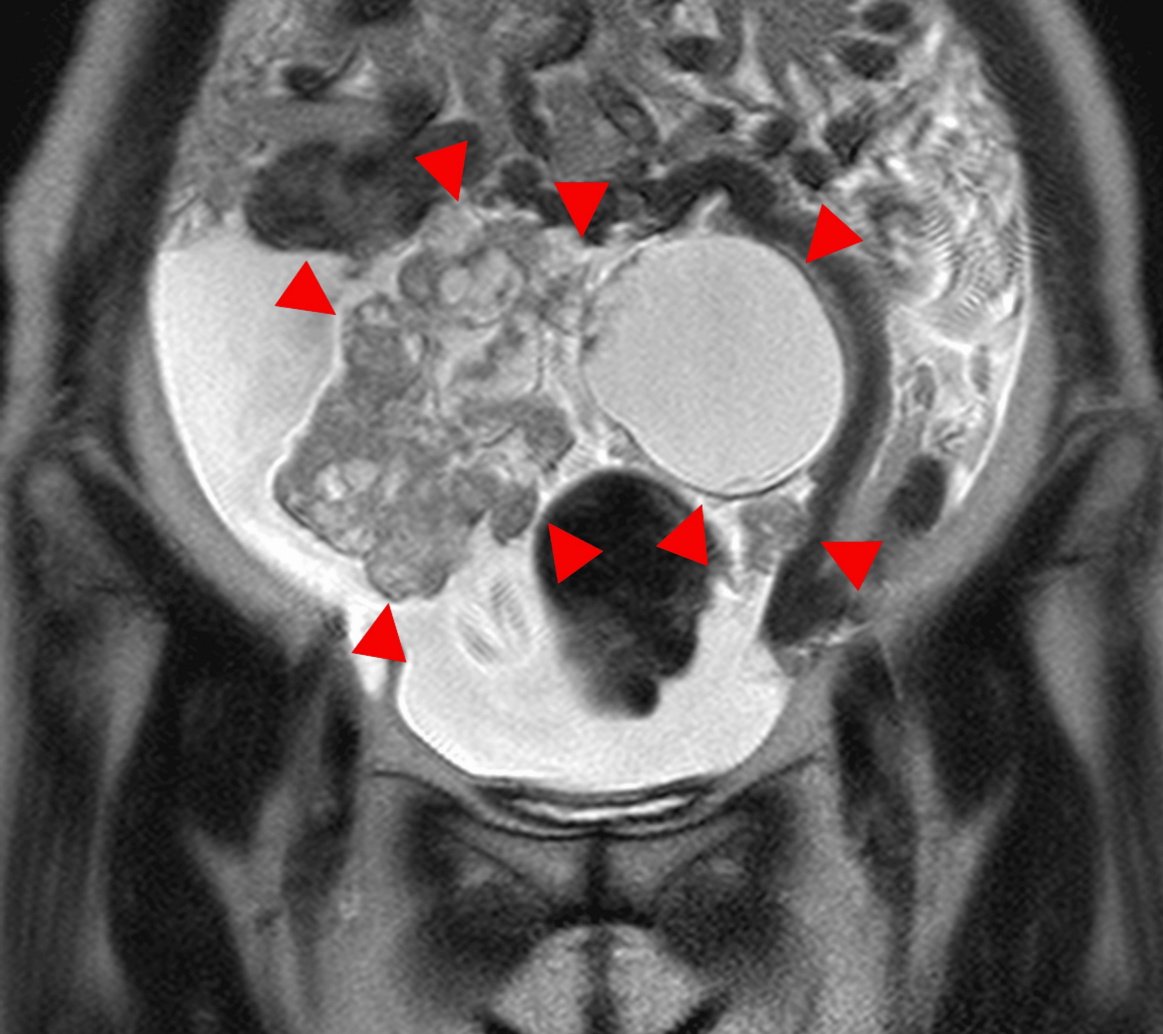
MRI of ovarian carcinosarcoma in a 61-year-old woman with a 17-cm tumor in the right ovary The coronal T2-weighted image shows a mass with an irregular margin, associated ascites, solid components, and cystic areas (arrowhead). The solid component demonstrates heterogeneous signal intensity.

The age of patients with ovarian carcinosarcoma ranged from 51 to 80 years (mean, 67.6 ± 8.8 years). The maximum tumor diameter ranged from 5.7 to 21.5 cm (mean, 11.6 ± 4.8 cm). Tumors were unilateral in 11 cases. Tumor morphology was lobulated in eight cases and ovoid in four cases. An irregular tumor margin was observed in six cases. All tumors contained both solid and cystic components. The solid component was nodular in all 12 cases. The solid component was predominant over the cystic component in seven cases, whereas the cystic component was predominant in five cases. On T2-weighted images, the solid component demonstrated heterogeneous low-to-high signal intensity in 11 cases. A stained-glass appearance with mixed high and low signal intensities within individual cysts was observed in two cases. The mille-feuille sign in the solid component was identified in two cases. Findings suggestive of hemorrhage, defined as high signal intensity on T1-weighted images and low signal intensity on T2-weighted images, were observed in six cases. Fluid-fluid levels were present in three cases. In all eight contrast-enhanced cases, the solid component showed heterogeneous nodular enhancement, with internal necrosis observed in one case. Lymphadenopathy was present in six cases, peritoneal dissemination in eight cases, and ascites in nine cases. Representative cases are shown in Figures 1-15. The mean ADC value of the solid component was 0.952 ± 0.186 × 10⁻³ mm²/s.

Imaging findings of endometrioid carcinoma are summarized in Table 1. Comparative analysis between ovarian carcinosarcoma and endometrioid carcinoma demonstrated statistically significant differences in age (67.6 years vs. 56.9 years, p=0.003), irregular margins (6/12:50% vs. 3/45:6.7%, p=0.002), predominance of the solid component over the cystic component (7/12:58.3% vs. 11/45:24.4%, p=0.037), nodular configuration of the solid component (12/12:100% vs. 25/45:55.6%, p=0.005), presence of the mille-feuille sign in the solid component (2/12:16.7% vs. 0/45:0%, p=0.041), and peritoneal dissemination (8/12:66.7% vs. 9/45:20%, p=0.004). We also summarized the inter-reader concordance rates and κ values for morphology and imaging findings in Table 2.

**Table 1:** Patient characteristics and MRI findings of ovarian carcinosarcoma and ovarian endometrioid carcinoma *Indicates a statistically significant difference (p<0.05) ADC, apparent diffusion coefficient; SD, standard deviation.

	Ovarian carcinosarcoma	Ovarian endometrioid carcinoma	p-value
Age (years), mean ± SD*	67.6 ± 8.8	56.9 ± 11.1	0.003
Maximum diameter (mm), mean ± SD	11.6 ± 4.8	11 ± 6.3	0.695
ADC value (×10^-3 ^mm^2^/sec, mean ± SD	0.952 ± 0.186	0.916 ± 0.185	0.632
	No. (%)	No. (%)	
Unilateral lesion (y/n)	11/1 (91.7)	39/6 (86.7)	>0.9999
Morphology			
Lobulated/oval	8/4 (66.7)	26/20 (57.8)	0.744
Irregular margin (y/n)*	6/6 (50)	3/42 (6.7)	0.002
Appearance			
Predominantly solid/cystic*	7/5 (58.3)	11/34 (24.4)	0.037
Internal heterogeneity on T2WI (y/n)	11/1 (91.7)	44/1 (97.8)	0.380
Stained-glass appearance (y/n)	2/10 (16.7)	10/35 (22.2)	>0.9999
Nature of solid components: nodular/papillary*	12/0 (100)	25/20 (55.6)	0.005
Mille-feuille sign (y/n)*	2/10 (16.7)	0/45 (0)	0.041
Hemorrhage/enhancement			
T1WI high and T2WI low intensities (y/n)	6/6 (50)	32/13 (71.1)	0.187
Fluid-fluid level (y/n)	3/9 (25)	10/35 (22.2)	>0.9999
Solid portion enhancement (y/n)	8/0 (100)	19/0 (100)	>0.9999
Internal heterogeneity on Gd-FST1WI (y/n)	7/1 (87.5)	16/3 (84.2)	>0.9999
Necrosis, nonenhance of the solid portion (y/n)	1/7 (12.5)	0/19 (0)	0.296
Accompanying findings			
Lymph node metastasis (y/n)	6/6 (50)	8/37 (17.8)	0.053
Peritoneal dissemination (y/n)*	8/4 (66.7)	9/36 (20)	0.004
Ascites (y/n)	9/3 (75)	25/20 (55.6)	0.325

**Table 2:** The inter-reader concordance rates and κ values for morphology and imaging findings of ovarian carcinosarcoma

	Concordance rate	κ values
Unilateral lesion	1	1
Morphology		
Lobulated	1	1
Oval	0.917	0.750
Irregular margin	1	1
Appearance		
Predominantly solid	1	1
Predominantly cystic	1	1
Internal heterogeneity on T2WI	1	1
Stained-glass appearance	0.833	0.000
Nature of solid components: nodular	1	1
Nature of solid components: papillary	1	1
Mille-feuille sign	0.833	0.000
Hemorrhage/enhancement		
T1WI high and T2WI low intensities	0.667	0.330
Fluid-fluid level	0.917	0.750
Solid portion enhancement	1	1
Internal heterogeneity on Gd-FST1WI	0.875	0.600
Necrosis, nonenhance of the solid portion	1	1
Accompanying findings		
Lymph node metastasis	0.917	0.750
Peritoneal dissemination	0.917	0.750
Ascites	0.917	0.750

## Discussion

In the present comparison between ovarian carcinosarcoma and endometrioid carcinoma, laterality, tumor shape, and the presence of hemorrhagic components did not differ significantly. However, irregular tumor margins, a solid-dominant pattern, and peritoneal dissemination show a significantly higher frequency in ovarian carcinosarcoma and thus may suggest this diagnosis rather than endometrioid carcinoma.

A review of 71 cases of ovarian carcinosarcoma reported a mean tumor diameter of 12 cm, with tumor laterality distributed as right-sided in 33 of 116 cases (28.4%), left-sided in 44 cases (37.9%), and bilateral in 35 cases (30.2%). Lymph node metastases were observed in 26 of 82 cases (31.7%), ascites in 61 of 99 cases (61%), and distant metastases in 56 of 76 cases (73.7%), most commonly involving the omentum (46.15%), peritoneum (40.38%), and fallopian tubes (28.85%) [1,2]. In the present cohort, the mean age was 67 years, the mean tumor diameter was 11.6 cm, and unilateral tumors were observed in 11 of 12 cases. Lymph node metastases and ascites were detected in 50% and 75% of cases, respectively, findings that are largely consistent with those of previous reports.

Only a limited number of case reports have described or illustrated detailed imaging findings of ovarian carcinosarcoma. Nomura et al. reported two cases: a 70-year-old woman with a 7-cm multilocular mass containing solid components and peritoneal dissemination, and a 47-year-old woman with a 12-cm mass with solid components; both patients underwent surgery followed by adjuvant therapy [10]. Itoh et al. reported a case in a 67-year-old woman with an 18-cm lesion showing thickened cyst walls and irregular intraluminal features with mixed cystic and solid components on MRI [11]. Ono et al. described a case in a woman in her 70s with bilateral ovarian masses containing mixed solid and multilocular cystic components on CT and MRI; the solid component of the right ovarian mass contained fat, showed heterogeneous signal intensity on T2-weighted images, diffusion restriction, and peritoneal dissemination, reflecting a liposarcomatous component [12]. Zheng et al. reported a 76-year-old woman with a 12.2-cm mass composed of mixed cystic and solid components, showing slightly low signal intensity on T1-weighted images, heterogeneous high and low signal intensity on T2-weighted images, and areas of low signal intensity on fat-suppressed T1-weighted images. The lesion demonstrated heterogeneous signal on DWI with an ADC value of 1.17 × 10⁻³ mm²/s, heterogeneous contrast enhancement, and associated ascites [13]. Jin et al. reported MRI findings in two patients aged 77 and 80 years with ovarian carcinosarcoma measuring 13.7 cm and 5.7 cm, respectively. In both cases, the tumors showed iso to high signal intensity on T1-weighted images and heterogeneous high signal intensity on T2-weighted images, reflecting cystic components with intratumoral hemorrhage and solid components. Diffusion restriction with low ADC values and marked heterogeneous enhancement in the early phase, with subsequent washout in the delayed phase, were observed and attributed to the admixture of epithelial and mesenchymal components [14].

Across these case reports, ovarian carcinosarcoma typically occurred in postmenopausal women and presented as a large (>10 cm) heterogeneous mass with mixed cystic and solid components, enhancing solid portions, and frequent intratumoral hemorrhage, necrosis, or peritoneal dissemination. The imaging findings in the present study are considered to be largely consistent with those previously reported.

A study evaluating CT and MRI findings in five cases of ovarian carcinosarcoma, with solid and cystic compositions, hemorrhage, necrosis, diffusion restriction, and enhancement patterns. All patients underwent CT, and two underwent MRI. The mean age was 45.4 years, and tumor diameters ranged from 11 to 14 cm. All tumors were unilateral (left ovary in four cases, right ovary in one case) and appeared as ill-defined solid and cystic masses. In the two cases evaluated by MRI, intratumoral hemorrhage and diffusion restriction in the solid components were observed, with ADC values ranging from 0.998 to 1.10 × 10⁻³ mm²/s. Moderate to heterogeneous enhancement and ascites were also reported [15]. Although the patients in that study were younger than those in the present cohort, several imaging features, including unilateral mixed solid-cystic masses, enhancement of solid components, diffusion restriction, and ascites, were consistent with our findings.

To our knowledge, no previous studies have compared imaging findings between ovarian carcinosarcoma and ovarian endometrioid carcinoma. Regarding endometrial carcinoma, studies comparing it with clear cell carcinoma have reported characteristic findings, such as multiple contiguous solid components extending along the cyst wall and broad-based solid nodules with a low height-to-width ratio [16,17]. In a study comparing endometrioid carcinoma and high-grade serous carcinoma, endometrioid carcinoma more frequently showed unilateral involvement (91.3% vs. 50.5%, p<0.001), larger tumor size (80.0% vs. 48.2%, p=0.005), an oval shape (64.0% vs. 17.3%, p<0.001), cyst-dominant masses with papillary protrusion (72.0% vs. 18.7%, p<0.001), cysts containing hemorrhagic components (82.6% vs. 4.3%, p<0.001), absent or minimal ascites (91.3% vs. 57.0%, p=0.002), and higher ADC values in solid components (0.979 ± 0.197 × 10⁻³ mm²/s vs. 0.820 ± 0.112 × 10⁻³ mm²/s, p=0.002) [18]. In contrast, another study evaluating the imaging-based differential diagnosis of ovarian malignancies noted that while no specific imaging findings for endometrioid carcinoma were identified, the frequency of peritoneal dissemination was significantly lower [19]. Although various imaging findings suggestive of endometrial carcinoma have been reported, accurate prediction of histological subtypes based solely on imaging findings is considered difficult due to significant overlap with other ovarian malignancies. In comparing ovarian carcinosarcoma with endometrioid carcinoma, no significant differences were observed in tumor laterality, shape, or the presence of hemorrhagic components. However, irregular tumor margins, predominance of solid components, and peritoneal dissemination were significantly more frequent in ovarian carcinosarcoma. These findings may support this diagnosis over endometrioid carcinoma.

Watanabe et al. evaluated the diagnostic significance of a “mille-feuille-like” layered appearance in ovarian carcinosarcoma (12 cases) and its differentiation from ovarian metastasis from colorectal carcinoma (18 cases) and primary ovarian carcinoma (40 cases). Compared with ovarian metastases from colorectal carcinoma, ovarian carcinosarcoma more frequently demonstrated intratumoral hemorrhage (p=0.02), irregular margins (p=0.048), unilateral involvement (p=0.02), and lower ADC values (p<0.01). A low ADC value (cutoff ≤0.87 × 10⁻³ mm²/s) showed a sensitivity of 66.7%, specificity of 94.4%, and accuracy of 81.0% for diagnosing ovarian carcinosarcoma [8]. The “mille-feuille-like” layered appearance was observed in 5 of 12 cases (41.7%) of ovarian carcinosarcoma and in 8 of 18 cases (44.4%) of ovarian metastases from colorectal carcinoma, but in none of the ovarian epithelial carcinoma cases (0%), with a significant difference (p<0.001). Although the frequencies of hemorrhage, layered appearance, and low ADC values differed between that study and the present study, the tendencies toward irregular margins and unilateral involvement were consistent. Variations in imaging finding frequencies may reflect differences in disease stage, patient characteristics, relative predominance of epithelial or mesenchymal components, imaging protocols, or scanner-related factors.

Saida et al. compared MRI findings between ovarian carcinosarcoma (12 cases) and high-grade serous carcinoma (30 cases). Tumor size was significantly larger in the ovarian carcinosarcoma group (mean, 13.6 cm vs. 9.0 cm; p=0.022). A “stained-glass-like” cystic appearance (67% vs. 23%, p=0.013), intratumoral hemorrhage (100% vs. 50%, p=0.003), necrosis (75% vs. 13%, p<0.001), and coexistence of endometriosis (33% vs. 7%, p=0.012) were significantly more frequent in ovarian carcinosarcoma, whereas ADC values and dynamic contrast enhancement patterns did not differ significantly between the groups [9]. Although the mean tumor size in the present study (11.6 cm) was comparable, the frequencies of a stained-glass-like appearance, hemorrhage, and necrosis were lower. However, both that study and the present study were limited by small sample sizes (12 cases each), and differences in component composition may substantially influence the observed imaging frequencies. Therefore, larger-scale studies are needed.

This study has several limitations. First, ovarian carcinosarcoma is a rare tumor, resulting in a small sample size and limited statistical power. Second, this was a retrospective, single-center study. Third, the extended study period resulted in inconsistencies in MRI field strength, scanner models, and imaging protocols, which may have affected the accuracy of ADC measurements. In addition, formal interobserver agreement analysis was not performed, and ADC measurements were not normalized across scanners. Fourth, ADC normalization across scanners is challenging, and future prospective studies with standardized protocols will be necessary.

## Conclusions

On MRI, ovarian carcinosarcoma tended to present as a mass with irregular margins, predominantly nodular solid components with partial cystic components, and a higher frequency of peritoneal dissemination. Understanding these results will help narrow the differential diagnosis of potentially nonspecific malignant ovarian tumors in the clinical setting, thereby performing effective treatment and improving prognosis. Further investigation with a larger number of cases and detailed correlation with histopathological findings is expected to improve the accuracy of preoperative diagnosis using MRI. 
